# Draft Genome Sequences of Four *Yersinia enterocolitica* Strains, Isolated from Wild Ungulate Carcasses

**DOI:** 10.1128/genomeA.00192-17

**Published:** 2017-04-13

**Authors:** Guerrino Macori, Angelo Romano, Daniela Adriano, Elisabetta Razzuoli, Daniela Manila Bianchi, Silvia Gallina, Alberto Bellio, Lucia Decastelli

**Affiliations:** a Food Control and Production Hygiene Unit, Istituto Zooprofilattico Sperimentale del Piemonte, Liguria e Valle d'Aosta, Turin, Italy; b Laboratory of Diagnostics, Istituto Zooprofilattico Sperimentale del Piemonte, Liguria e Valle d'Aosta, Genova, Italy

## Abstract

This study describes the draft genome sequences of four *Yersinia enterocolitica* strains, originally isolated from ungulate carcasses. These isolates were typed biochemically and two were determined to be highly virulent (biotype 1B). The draft genome sequences had a mean size of 4.77 Mb and a mean G+C content of 47.1%.

## GENOME ANNOUNCEMENT

*Yersinia enterocolitica* is a Gram-negative bacterial pathogen and the causative agent of yersiniosis in humans (1). Infection is frequently associated with the consumption of contaminated foods (2), including pork, which is considered to be the main source of the pathogen (3), beef, cow’s milk, and poultry, as well as raw vegetables (4). Based on their biochemical properties, pathogenic strains of virulent *Y. enterocolitica* spp. can mainly be differentiated from nonvirulent ones (5, 6). The strains presented here were isolated originally from the carcasses of three wild boars and one fallow deer and were biochemically typed: two of them were identified as 1B (highly virulent), and two were identified as 1A (nonvirulent).

Bacterial genomic DNA was extracted using the DNeasy blood and tissue kit (Qiagen, USA). The genomic libraries were prepared using the Nextera XT library prep kit, and 250-bp paired-end sequencing was performed using the Illumina MiSeq platform (Illumina, USA). Raw reads were preprocessed using Trimmomatic version 0.36 software (7) to remove adapter sequences and low-quality reads. *De novo* assembly was performed using the SPAdes version 3.9.1 genome assembler (8). Assemblies ranging between 34 and 86 contigs were generated per genome with total sizes ranging from 4.71 to 4.79 Mb (mean, 4.77 Mb) and a mean G+C content of 47.1%.

Genome annotation with Prokka (8) identified potential protein-coding sequences (CDSs), as well as the number of rRNAs and tRNAs for each strain. The CDSs include predicated virulence factors present in the genus *Yersinia* (http://www.mgc.ac.cn/cgi-bin/VFs/compvfs.cgi?Genus=Yersinia) and encompass genes associated with flagellar biosynthetic proteins, adhesion, invasion, secretion system apparatus, and other virulence factors that characterize any strain.

The draft genome sequences were annotated using the NCBI GenBank annotation pipeline and RAST genome annotation server (9). The number of CDSs per genome ranged from 2,886 to 3,096, accounting for 89.6% to 90.6% of the genome. The *N*_50_ values for the strains are listed in Table 1. For two strains, Y2wildboar1B and Y4wildboar1A, multilocus sequence types were not determined. Strains Y1wildboar1B and Y4wildboar1A were assigned sequence types (ST) ST114 and ST15, respectively.

**TABLE 1:** Genome assembly details and statistics

Strain	NCBI BioSample no.	GenBank accession no.	Genome size (bp)	No. of contigs	*N*_50_ (bp)
Y1wildboar1B	SAMN06187705	MTCV00000000	4,782,007	86	178,496
Y2wildboar1B	SAMN06187706	MTCW00000000	4,790,440	84	283,617
Y3wildboar1A	SAMN06187707	MTHH00000000	4,783,238	34	411,957
Y4fallowdeer1A	SAMN06187708	MTHI00000000	4,710,674	53	376,163

### Accession number(s).

This whole-genome shotgun project has been deposited in GenBank under the accession numbers listed in Table 1. The versions described in this paper are the first versions.
